# COVID-19 from the perspective of urban and rural general adult mental health services

**DOI:** 10.1017/ipm.2020.62

**Published:** 2020-05-21

**Authors:** J. Lyne, E. Roche, M. Kamali, L. Feeney

**Affiliations:** 1 Wicklow Mental Health Services, Newcastle Hospital, Greystones, Co., Wicklow, Ireland; 2 Royal College of Surgeons in Ireland, 123 St. Stephen’s Green, Dublin 2, Ireland; 3 Cluain Mhuire Community Mental Health Services, Newtownpark Avenue, Blackrock, Co., Dublin, Ireland

**Keywords:** COVID-19, general adult psychiatry, innovation, service development

## Abstract

COVID-19 has presented society with one of the greatest challenges in living memory. Community Mental Health Teams (CMHTs)have needed to adapt quickly to a rapidly developing situation which has had a dramatic impact on society. In this piece, we describe some of the early challenges for CMHTs within two mental health services based in Dublin and Wicklow. We also discuss ongoing developments and anticipate the need for further vigilance as the COVID-19 pandemic continues to evolve.

## Introduction

“*There is no time for ease and comfort. It is time to dare and endure*”. Winston Churchill

COVID-19 is the greatest global pandemic experienced in over a century, and it has impacted on a range of physical, mental and social health indices (WHO, 2020). The most acute concern has been for the physical health needs of the population; as of 16 April 2020, there were 1 995 983 confirmed cases and 131 037 confirmed deaths related to COVID-19 infections internationally (WHO, 2020). The capacity of medical hospitals to deal with the pandemic has been thoroughly overwhelmed in many countries.

In addition to the associated physical morbidity and mortality, it is recognised that population mental health may be adversely affected in many ways during a pandemic (Pfefferbaum and North, 2020). The emotional trauma of experiencing a severe life-threatening respiratory illness, the grief associated with increased death rates and the long-term psychological sequelae of quarantine are just some examples of how mental health may be adversely effected (Brooks *et al.*, 2020). These mental health difficulties may be exacerbated in those already vulnerable and may also arise *de novo* among the general population (Cullen *et al.*, 2020). A potential economic recession could further compound the impact on population mental health, with possible increase in mental disorders, substance abuse and suicidal behaviours (Frasquilho *et al.*, 2016).

What then, has been the effect on general adult mental health services during the COVID-19 pandemic? It is clear that the demand for psychiatric healthcare has remained, and may increase, during the acute phase and recovery phase of the pandemic. Community mental health teams (CMHTs) have needed to adapt to challenges associated not only with the pandemic but also with public health measures taken to control the pandemic. In particular, the challenges arising from social distancing recommendations have been profound in a medical specialty that is underpinned so fundamentally by the need to develop and maintain a therapeutic relationship. CMHTs have needed to quickly adapt their model of care more rapidly than any other time in living memory. The premature mortality and poorer physical health status associated with mental disorder recognised prior to the COVID-19 pandemic (Rodgers *et al.*, 2018) further complicates the vital need to balance physical and mental health needs of service users.

The COVID-19 pandemic has, at least temporarily, transformed how we deliver care in a community mental health setting. The national public health management plan is evolving in response to this pandemic, and similarly, CMHTs will need to respond in parallel. Here, we discuss some of the challenges and developments in the delivery of general adult mental healthcare arising in the context of the COVID-19 pandemic.

## Early planning and communication

The adult mental health services within which the authors work (Cluain Mhuire Mental Health Services in Dublin and Newcastle Mental Health Services in Wicklow) responded to identified needs early in the COVID-19 outbreak. One of the first notable changes was an increased frequency of senior management meetings, which was invaluable in developing a coordinated response to the myriad of complex issues presenting. The Health Service Executive (HSE) Executive Management Team (including Clinical Directors, Executive Clinical Director, heads of disciplines, head of service and service managers) met daily initially, including at weekends and a bank holiday up until April, and meeting then tapered down to three per week in May. There were also several other senior groups in each hospital meeting several times per week, and a dedicated COVID management team commenced in one of the hospital. Clear and reliable information from this early planning was disseminated across all tiers of the service through multi-clinical meetings to effect the necessary changes.

An early priority was to develop a coherent service delivery plan with contingencies to help navigate challenges that may arise. This necessitated analysis of the service and the identification of elements that were absolutely essential to maintain: inpatient services, residential services, emergency assessments, acute day hospitals, medication delivery and monitoring. The analysis of the service was conducted by local senior clinicians, including Clinical Directors, heads of disciplines and senior management who were assisted by the local Consultant Psychiatrists. The analysis was documented in excel spreadsheets, which included emergency contingency planning for various scenarios of COVID-19 infections in the service. One of the core aims was to minimise the risk of COVID-19 infection or transmission for both service users and staff within the service. Therefore, minimising face to face contact between people was desirable, or at the minimum ensuring social distancing for essential face to face contacts. In practice, for staff, this meant taking such measures as avoiding shared office space, working from home where possible and substituting in-person meetings with videoconferencing, telephone or email contact. It also meant risk assessing all of our community locations with regard to minimising infection transmission.

At-risk groups among staff members and service users were identified early and prioritised in service changes. Staff were trained in the use of personal protective equipment (PPE), amid concerns in relation to its availability (Emanuel *et al.*, 2020), and were also directed to update their training in effective hand hygiene. Supplies of hand sanitiser, PPE and educational materials were secured. Contingency planning for how to manage cases of COVID-19 among staff and service users was developed and updated. Staffing rosters were amended and bolstered with backups identified in case of illness. Redeployment protocols were established for staff not normally working in inpatient or residential settings. Videoconference and telemedicine facilities within the service were developed, in line with international commentary and recommendations (Whaibeh *et al.*, 2020; Royal College of Psychiatrists). There were some understandable delays in moving towards a telemedicine service, and working from home, as the necessary equipment was not available to all clinicians at the outbreak of the pandemic. Telephone consultation was utilised while services were planning the use of telemedicine, and face to face contact with service users occurred when necessary. One of our services had the advantage of an electronic patient record that was accessible from home. This increased the potential for some staff in this service to work from home where possible.

## Inpatient and residential mental health settings

Inpatient and residential mental health settings, such as vulnerable hostel residents and those living in a supported living arrangement, posed multiple complex challenges for safely reducing the risk of COVID-19 transmission. As might be expected, delusions, obsessions and other psychopathology have sometimes had themes related to pandemic-related fears. By their nature, general adult inpatient units provide care to people with a diverse range of clinical presentations, including service users who may not have the capacity to adhere to important guidelines such as social distancing. With this in mind, all non-essential visits to the inpatient unit were discontinued, along with non-essential leave for service users. Changes designed to reduce infection transmission included staggering ward visits, reducing the number of clinicians from each CMHT involved with inpatient care and virtual multidisciplinary ward rounds.

Consideration of COVID-19 transmission within the inpatient unit became a factor affecting clinical decision-making for new hospital admissions, delivery of inpatient care and discharge planning. In many cases, careful consideration of relative risks was required in relation to whether, and when, to admit or safely discharge from the inpatient unit. The inpatient units accessed by both of our adult mental health services have shared rooms, although provision was made for isolation rooms in each facility. These were reserved for individuals at risk of COVID-19 due to travel or contact with a case and for those with possible symptoms of COVID-19 illness. Electroconvulsive Therapy (ECT) which is an aerosol-generating procedure had to be suspended initially while safe protocols were developed and the necessary PPE was obtained. Another occasional challenge was the safe use of physical restraint for highly agitated or aggressive service users.

Specific medicolegal implications arose in the context of COVID-19 not only relating to the Mental Health Act 2001 but also the Health (Preservation and Protection and other Emergency Measures in the Public Interest) Act 2020. There was consultation between public health services and adult mental health services in relation to the detention and isolation of patients under the Health Act 2020, a process which is ordered by medical officers rather than consultant psychiatrists. The definition of *‘*serious and immediate harm*’* under Section 3 (1) (a) of the Mental Health Act 2001 was also subject to discussion, specifically as to whether this could include the risk of transmission of COVID-19. It was decided that COVID status would not generally be relevant to Section 3 (1) (a) and that existing legislation should not change. Furthermore, it was made clear that seclusion was not an appropriate response for managing a COVID positive service user who would not adhere to isolation guidelines. The approach utilised was to encourage people to adhere to recommendations and to work as closely as possible with service users, sometimes relaxing rules regarding smoking and phone access to achieve this.

Changes were introduced by the Mental Health Commission for performing independent psychiatric assessments among certain categories of patients detained under the Mental Health Act 2001, as well as to the operation of Mental Health Tribunals. The interview aspect of Section 17 assessments was changed to telephone assessment, while Mental Health Tribunals are now attended remotely. Balancing the physical health and mental health needs of inpatient service users while upholding due legal process can be a complex matter, and a degree of flexibility and adaptation has been required on the part of clinicians and services users alike for this to occur effectively.

## Outpatient settings

Face to face contacts were dramatically reduced following the COVID-19 onset. Teleconference facilities were organised within services and teams, and smartphone and laptops were organised for all medical staff and many other clinicians. Phone and video assessments and consultations were dramatically increased. The intervals between injections of long-acting antipsychotic medications were stretched where possible and extra supplies of clozapine were secured to enable delays in blood testing where necessary. Efforts were made to reduce home visits for delivery of Clozapine and long-acting injectable medication; however, this proved a challenge in some rural areas where public transport is limited. Essential face to face meetings proceeded using PPE where indicated and perspex screens were organised in consultation rooms and other settings. Movement of staff between different residential units was stopped and visiting was restricted. Multidisciplinary meetings were arranged through video or teleconference facilities which proved reliable and effective. Medical, nursing and allied health professionals worked from home where possible using phone or video consultation with service users.

All face to face groups were cancelled in the services, and delivery of day hospital and home treatments were reduced as much as possible unless delivery of such treatments could assist in preventing a hospital admission. Delivery of face to face assessments and treatments such as occupational therapy was restricted on the inpatient unit. Psychology, occupational therapy, social work and family work in the community were continued using phone contact or utilisation of teletherapy by some clinicians. The Dialectical Behaviour Therapy group in one of the services continued running groups and individual sessions using Zoom within weeks of the lockdown commencing. In these early phases of COVID-19, CMHTs did not notice major impact due to the closure of secondary services, such as counselling in primary care and Jigsaw, however, this impact is certainly anticipated for the coming months should there be ongoing decreased delivery of these services.

A central phone line was organised to make it easier for General Practitioners to obtain urgent advice and assessments when needed, with a particular focus on keeping these assessments in the community where possible. The central phone line implemented by one of the hospitals was manned by nursing staff who had immediate phone contact available with the Registrar and the catchment area Consultant or Consultant on call after working hours. When General Practitioners made contact, clinical advice was given, and where immediate assessment was needed, efforts were made to organise an emergency outpatient appointment on that day, or within 48 hours if appropriate. Unscheduled walk-in presentations to inpatient units were discouraged. In general, service attenders, their families and others were very understanding of the need for a dramatic change in the delivery of services which made the reorganisation of service delivery much easier. Our services do not cover an Emergency Department which would in itself require a significant planning strategy for COVID-19.

## Rural-specific issues

A significant part of the catchment area is in a rural setting which itself provided a distinct set of challenges. Some rural settings are poorly connected to broadband and have poorer mobile phone connectivity. This increases the challenge of conducting assessments using various technologies such as Zoom and Blueye. Phone consultations were often used in this instance, and where necessary face to face assessments using precautions of COVID-19 symptom screening, perspex screens and masks were indicated. The increase in use of technology could be of great benefit to rural settings in future if adequate technology can be developed, particularly for isolated individuals with limited travel options.

## Challenges in transitions

There were many challenges to the implementation of the changes outlined above. For example, it was not possible to eliminate all face to face contact. Crisis presentations were seen by each CMHT on most weeks, and usually, there were several crisis presentations each week. Where admission to hospital was a possibility, there was often a face to face assessment with service users to reduce this likelihood. It was deemed important to limit admission to the inpatient unit as much as possible due to risk of bringing COVID-19 into a vulnerable setting. Disseminating information to General Practioners in relation to how to access help without presenting to the acute unit required collaboration with local General Practice groups.

Initially, it was challenging to implement social distancing, and efforts were made to create a culture of social distancing to assist with this. The number of inpatient beds was reduced to facilitate social distancing in this environment; however, in spite of efforts by inpatient staff, it remained difficult to ensure social distancing for vulnerable individuals with severe illness who may lack capacity. The heads of each discipline and Consultants regularly discussed the importance of social distancing and frequent handwashing at weekly Multidisciplinary Team (MDT) phone meetings, and several emails were sent around to all staff in the service.

Further challenges included frustration among staff at times with new ways of working due to the limitations imposed by COVID-19 restrictions. For example, it was difficult to plan leave during this time as we were uncertain when we may require extra staff due to leave occurring in the context of a COVID-19 positive case among staff or patients. Furthermore, some staff did not want to take leave due to the lockdown.

Most of these challenges were addressed using very frequent meetings among senior staff, usually utilising teleconferencing or videoconferencing. The multidisciplinary nature of the committees and meetings proved essential for implementing several of the changes needed. Meetings within teams and within disciplines were also helpful for disseminating information about COVID management. National meetings among Clinical Directors and Executive Clinical Directors were also hugely beneficial for sharing knowledge and experiences. Increased email contact between all stakeholders ensured that up to date information flowed freely between stakeholders.

## Ongoing challenges for CMHTs

The effects of COVID-19 continue to unfold and the future remains uncertain. There is still much to consider from a clinical and service development perspective. Ongoing development of telemedicine is needed now more than ever given the potential for the current pandemic to remain a long-term risk and indeed to plan for any future pandemics. The academic teaching programme was continued effectively using Zoom technology in one of the services, although the safety of delivering any clinical work using technology needs ongoing assessment at senior management levels.

Developing strategies for identification of vulnerable individuals who may be at risk of not seeking help due to COVID-19 must also be considered. For example, delivering information sheets to all service attenders with instructions of how to obtain help during the COVID-19 crisis should be considered, and such information could include advice that the importance of seeking help for mental distress remains as important as ever. There are also challenges for individuals who have poor transport links or poor connectivity for telemedicine particularly in rural regions, and increased efforts to maintain contact is needed for these groups.

It is widely anticipated that there will be a surge of referrals to CMHTs in the medium term and in the aftermath of COVID-19. Furthermore, existing service attenders may be at increased risk of presenting with relapse. With this in mind, we are increasing efforts to reduce waiting lists at present by continuing to assess new referrals. Increase in demand may later require increased resourcing; however, this is currently difficult to predict as we enter unchartered waters.

Leadership within mental health teams is needed now more than ever to combat much of the challenges posed by COVID-19. Ensuring that adequate resources are provided for training and materials such as PPE are essential (Shi *et al.*, 2020). Furthermore, intangible elements such as trust between and within teams and positive working environments can prove invaluable during difficult times. With this in mind, ensuring that our own mental health is cared for would be a prudent approach. It has been well documented that the mental health of frontline healthcare staff is essential at this time (Gavin *et al.*, 2020), and it is important to recognise that delivering healthcare within intense environments could have mental health impacts for workers.

There is currently great uncertainty in relation to how COVID-19 restrictions will evolve in the coming months, and even years. While the current measures have been implemented to respond to the acute issues that have arisen, it is unclear whether the current model of clinical delivery will be sustainable should restrictions continue for the foreseeable future. Furthermore, the more limited access to mental health services may become unacceptable to service users over time. There is a need to plan for possible alternative models of care delivery, given the potential duration of this crisis.

We should now also be considering how the mental health of the population will be affected following resolution of the COVID-19 crisis (Das, 2020). Anticipating challenges will ensure that we can continue to plan for effective intervention in this rapidly evolving situation. It is possible that many individuals are not seeking help at present due to concerns about contracting COVID-19 while attending healthcare facilities (Kelly, 2020). We must learn from strategies employed by other psychiatric services globally, ensuring that we share ideas for combating this great challenge (Shao *et al.*, 2020). It is also crucial that we are not distracted by excessive media coverage and possible increased anxiety due to false news about COVID-19 (Lancet, 2020).

On a more optimistic note, there may be some unexpected benefits from COVID-19 in the long term such as an increase in access to telemedicine which could have many potential uses in CMHTs (Whaibeh *et al.*, 2020). Increased governance planning may strengthen team spirit, and if we can harness this in a positive manner, we may be better equipped to deal with any subsequent increase in population mental illness (Xiang *et al.*, 2020).

It is essential that we continue to follow advice of reputable organisations such as the Health Service Executive and the World Health Organisation in our ongoing management of COVID-19 (WHO, 2020). While we need to continue trusting our experts, we must also deliver national and international guidance in a local context by continuing to apply clinical governance locally to ensure the safety of our patients and staff. There remains much to fight for in the battle against COVID-19 and we will remain vigilant in the time ahead as the COVID-19 pandemic evolves. CMHTs will undoubtedly have an important role to play in the coming months as society recovers from the scars that have been dealt by the COVID-19 pandemic (Goldberg, 2020).

## Financial support

This article received no specific grant from any funding agency, commercial or not-for-profit sectors.

## Conflict of interest

The authors have no conflict of interest to disclose.

## Ethical standards

The author asserts that all procedures contributing to this work comply with the ethical standards of the relevant national and institutional committee on human experimentation with the Helsinki Declaration of 1975, as revised in 2008. The authors assert that ethics committee approval was not required for this perspective piece.
